# A comparison of a ketogenic diet with a LowGI/nutrigenetic diet over 6 months for weight loss and 18-month follow-up

**DOI:** 10.1186/s40795-020-00370-7

**Published:** 2020-09-24

**Authors:** Maria Vranceanu, Craig Pickering, Lorena Filip, Ioana Ecaterina Pralea, Senthil Sundaram, Aseel Al-Saleh, Daniela-Saveta Popa, Keith A. Grimaldi

**Affiliations:** 1 Department of Toxicology, Iuliu Hatieganu University of Medicine and Pharmacy, Cluj Napoca, Romania; 2 Institute of Coaching and Performance, School of Sport and Wellbeing, University of Central Lancashire, Preston, UK; 3 Department of Bromatology and Hygiene, Iuliu Hatieganu University of Medicine and Pharmacy, Cluj Napoca, Romania; 4 Department of Nutrigenetics and Personalized Nutrition, Eurogenetica, Rome, Italy; 5 Prenetics Ltd, Quarry Bay, Hong Kong SAR China; 6 Arab Gulf University, Manama, Bahrain; 7 Prenetics DNAfit Research Centre, London, UK

**Keywords:** Glycaemic index, Genetic testing, Nutrigenetics, Weight loss, Ketogenic, BMI, Cholesterol

## Abstract

**Background:**

Obesity and its related metabolic disturbances represent a huge health burden on society. Many different weight loss interventions have been trialled with mixed efficacy, as demonstrated by the large number of individuals who regain weight upon completion of such interventions. There is evidence that the provision of genetic information may enhance long-term weight loss, either by increasing dietary adherence or through underlying biological mechanisms.

**Methods:**

The investigators followed 114 overweight and obese subjects from a weight loss clinic in a 2-stage process. 1) A 24-week dietary intervention. The subjects self-selected whether to follow a standardized ketogenic diet (*n* = 53), or a personalised low-glycemic index (GI) nutrigenetic diet utilising information from 28 single nucleotide polymorphisms (*n* = 61). 2) After the 24-week diet period, the subjects were monitored for an additional 18 months using standard guidelines for the Keto group vs standard guidelines modified by nutrigenetic advice for the low-Glycaemic Index nutrigenetic diet (lowGI/NG) group.

**Results:**

After 24 weeks, the keto group lost more weight: − 26.2 ± 3.1 kg vs − 23.5 ± 6.4 kg (*p* = 0.0061). However, at 18-month follow up, the subjects in the low-GI nutrigenetic diet had lost significantly more weight (− 27.5 ± 8.9 kg) than those in the ketogenic diet who had regained some weight (− 19.4 ± 5.0 kg) (*p* < 0.0001). Additionally, after the 24-week diet and 18-month follow up the low-GI nutrigenetic diet group had significantly greater (p < 0.0001) improvements in total cholesterol (ketogenic − 35.4 ± 32.2 mg/dl; low-GI nutrigenetic − 52.5 ± 24.3 mg/dl), HDL cholesterol (ketogenic + 4.7 ± 4.5 mg/dl; low-GI nutrigenetic + 11.9 ± 4.1 mg/dl), and fasting glucose (ketogenic − 13.7 ± 8.4 mg/dl; low-GI nutrigenetic − 24.7 ± 7.4 mg/dl).

**Conclusions:**

These findings demonstrate that the ketogenic group experienced enhanced weight loss during the 24-week dietary intervention. However, at 18-month follow up, the personalised nutrition group (lowGI/NG) lost significantly more weight and experienced significantly greater improvements in measures of cholesterol and blood glucose. This suggests that personalising nutrition has the potential to enhance long-term weight loss and changes in cardiometabolic parameters.

**Trial registration:**

NCT04330209, Registered 01/04/2020, retrospectively registered.

## Background

Obesity is characterised by excessive fat accumulation, and it is well established that the percentage of the population that is either obese or overweight is rising over time [1]. Obesity is also associated with several health issues, including the development of metabolic syndrome, hypertension, cardiovascular disease, arthritis, and various cancers [2]. The causes of obesity are not simply the consumption of a greater amount of energy than is utilised; instead, obesity is a complex disorder, with many biological, psychological, and sociological factors combining in its development [3].

A considerable number of health interventions have been trialled to reduce obesity [2]. In terms of dietary interventions, the efficacy of these trials is mixed, with a sizeable proportion of dieters regaining more weight than they initially lose [4]. Emerging research suggests that the ability to place obese subjects on a personalised nutrition regime using genetic information may increase both adherence to that diet and subsequent post-diet nutrition, enhancing health outcomes [5–8]. This has been demonstrated in recent years in studies on gene-diet interactions and the emergence of nutrigenetics, a goal of which is to add a level of personalization to standard nutrition by adjusting it according to genetic variation. Prior studies suggest that specific genetic variants may increase susceptibility to certain disease states, but that this increased risk can be reduced or completely mitigated with dietary modification [9–12]. As an example, variation in *MTHFR* C677T genotype leads to alterations in plasma folate status [13], which can increase the risk of hyperhomocysteinemia, potentially increasing the risk of cardiovascular disease [14]. However, in those with the risk (TT) genotype, intakes of greater than the recommended daily allowance (RDA) of folate are associated with a lowering of homocysteine to reference values [9]. This indicates that a “one-size fits all” approach to nutrition is perhaps insufficient, and that individualised nutrient guidelines may improve population health.

Whilst obesity itself increases all-cause mortality [15], it is also associated with other issues that may negatively affect health, including elevated total cholesterol (TC) [16], lower high-density lipoprotein cholesterol (HDL) [17] and raised fasting blood glucose (FBG) [18]. Management of these markers is important in optimising individual health and reducing mortality risk.

A key observation, made by many, is the unsurprising conclusion that hypocaloric dietary adherence, regardless of the diet macronutrient composition, is the most critical aspect of weight loss success [19–21]. Furthermore, ongoing adherence to healthy nutrition after the initial caloric restriction phase is equally critical to achieving long-term weight loss. A weight management study by Arkadianos et al. [5] utilised a nutrigenetic test which was not specifically targeted at weight loss. Participants were allocated into either a nutrigenetic or control group, similar in calories and macronutrient composition. Within the nutrigenetic group, diet was modified by the addition of certain micronutrients based on the genetic results, and small changes in macronutrients (e.g. a reduction in refined carbohydrate from a maximum of 10% calories to max 8% calories; reduction of glycaemic load (GL) (from a maximum of 100 to 70). Adherence was determined by clinic visits and questionnaires. Weight loss during the 24-week diet was similar in both groups, but during follow-up over a 1-year period, the nutrigenetic group maintained weight loss while the non-genetic group regained weight [5].

The aim of the present study was to observe weight loss 2-stage process:

1. A 24-week dietary intervention comparing two diets: The well-known, and generally the most effective in the short-term ketogenic diet [22] vs. A lowGI/Nutrigenetic diet.
2. A second phase, lasting 18 months, utilising standard guidelines for the ketogenic group vs standard guidelines modified by nutrigenetic advice for the lowGI/NG group.

The study protocol was similar to Arkadianos et al. [5] and used a nutrigenetic test which had been further developed in the European Union (EU) funded consortium project EUROGENE [23]. The addition of nutrigenetic advice was not designed nor proposed to patients as a weight loss diet, nor to predict either disease risk or obesity risk; the aim was to optimize the nutrient content of an individual’s daily food intake, based on current understanding of an individual’s genetic profile. Whilst an individual is achieving weight loss, food consumption is generally reduced and some nutrients in the diet may not be in adequate supply; nutrigenetics may be a tool to help achieve optimum nutrient content on an individual basis. Furthermore, the use of nutrigenetics in designing personalized diet and lifestyle programs has the potential to increase motivation and compliance with long-term lifestyle changes.

The primary objective was measurement of weight loss in the two groups after the 24-week diet and at 18-months of follow up. Secondary objectives were blood measurement of key risk factors, comprised of glucose, total cholesterol, and HDL cholesterol.

## Methods

One hundred fourteen subjects (1 overweight; 113 obese) subjects (M = 55, F = 59, age 24-56y, all of Romanian heritage and similar socio-economic status), who were patients at a weight management clinic (Bucharest, Romania), gave written informed consent for their weight loss data to be prospectively analysed for this study. All patient data were handled according to the Romanian Code of Medical Deontology and in accordance with the Helsinki Agreement. Approval was given by the Ethics Committee of the University and Pharmacy, Cluj Napoca, Romania (registration number 444). Upon enrolment at the weight management clinic, the subjects self-selected either a ketogenic diet or a low-GI nutrigenetic diet. A ketogenic diet was utilised as the comparison diet due to its reported efficacy in the treatment of obesity [24]. Fifty-three subjects (25 female; age 43.0 ± 7.2y) selected the ketogenic diet plan, and 61 subjects (34 female; age 42.0 ± 6.7y) selected the low-GI nutrigenetic diet plan. Subjects in the low-GI nutrigenetic diet group underwent DNA testing (NutriGENE by Eurogenetica Ltd./DNAfit, UK) for 28 Single Nucleotide Polymorphisms (SNPs) in 22 genes with good evidence of gene-diet/lifestyle interactions (Table 1). Overall participation in both diet groups cost a similar amount, comprised of approximately €300 for the genetic test along with 1-month diet plan, initial evaluation, body composition, and medical history for the nutrigenetic group, and €280 for the ketogenic group, providing Ketostix and the same evaluations. Further visits through the 24-week program the overall cost per patient was approximately €800. After 24 weeks diet follow up visits had no further cost, After the diet period, the subjects were monitored for an additional 18 months.

**Table 1:** Gene and polymorphisms tested in the low-GI nutrigenetic patient group

*Gene*	*Polymorphism*	*Reference Allele Freq*	*Heterozygote*	*Alt*	*MAF*
*ACE* [25, 26]	Ins/Del (rs4646994)	25%	54%	21%	48%
*ADRB2* [27, 28]	*Arg16Gly (rs1042713)*	*43%*	*39%*	*18%*	*38%*
*ADRB2* [27, 28]	Gln27Glu (rs1042714)	44%	44%	11%	34%
*APOC3* [29]	C3175G (rs5128)	69%	25%	7%	19%
*APOA2* [30, 31]	-265 T > C (rs5082)	28%	44%	28%	50%
*CAT* [32]	C-262 T (rs1001179)	56%	41%	3%	24%
*CYP1A2* [10, 33]	-163A > C (rs762551)	43%	41%	16%	37%
*EPHX1* [34, 35]	Tyr113His (rs1051740)	51%	38%	11%	30%
*FABP2* [36]	Ala54Thr (rs1799883)	54%	43%	3%	25%
*FTO* [30, 37]	A/T (rs9939609)	21%	52%	26%	48%
*GPX1* [38]	Pro198Leu (rs1050450)	57%	43%	0%	21%
*GSTT1* [39, 40]	Ins/Del	61%	39%		
*GSTM1* [39, 40]	Ins/Del	28%	72%		
*HLA-DQ* [41]	rs2395182_DQA1201	93%	7%	0%	3%
	rs7775228_DQB1202	72%	28%	0%	14%
	rs2187668_DQ25	74%	26%	0%	13%
	rs4639334_DQ7	72%	20%	8%	18%
	rs4713586_DQ4	100%	0%	0%	0%
	rs7454108_DQ8	89%	11%	0%	6%
*IL6* [42, 43]	G-174C (rs1800795)	49%	41%	10%	30%
*LCT* [44]	−13,910-CT (rs4988235)	53%	42%	5%	26%
*LPL* [45]	C1595G (rs328)	74%	23%	3%	15%
*MTHFR* [9, 13]	C677T (rs1801133)	33%	49%	18%	43%
*PPARG* [46, 47]	Pro12Ala (rs1801282)	84%	15%	2%	9%
*SOD2* [12]	C-28 T (rs4880)	26%	54%	20%	47%
*TCF7L2* [11, 48]	C/T (rs7903146)	52%	43%	5%	26%
*TNF* [49, 50]	G-308A (rs1800629)	62%	36%	2%	20%
*VDR* [51, 52]	C > T (taq1) (rs1544410)	39%	38%	23%	42%

At the study onset, patients were not type 1 or type 2 diabetics, although many were hyperglycemic, a common issue in obese subjects. Any patients with records of any other disease were excluded prior to commencing the dietary intervention. The patients, apart from obesity, were otherwise “healthy”. According to the Clinical Guidelines on the Identification, Evaluation, and Treatment of Overweight and Obesity in Adults [53], an obese person is considered healthy if they present with fewer than three of these conditions:

- a waist measurement over 40 in. in men or over 35 in. in women
- triglyceride levels in the blood of > 200 mg per deciliter (mg/dl)
- levels of high-density lipoprotein below 35 mg/dl
- fasting blood glucose > 120 mg/dl
- blood pressure ≥ 14/90 mmHg

### Diet overview

Both diets were followed for 24 weeks and contained approximately1600kcal per day. Both groups were provided with a meal plan and nutritional advice by the lead study author, a qualified nutritionist, a position requiring at least a Masters degree. After the 24-week study period, the subjects were monitored for an additional 18 months. Here, the ketogenic group followed population based nutrition and exercise guidelines, whilst the low-GI nutrigenetic group followed population based guidelines, slightly modified based on the genetic results of each patient. Other than the modifications to the standard diet and exercise program, the patients in both groups were treated in an identical manner.

### Ketogenic diet

The ketogenic diet group were instructed to consume ≤35 g of carbohydrates per day, and ≤ 10% of total calories were from saturated fats. Daily protein intake was set at 1.2 g/kg bodyweight for females, and 1.5 g/kg bodyweight for males. A sample day’s menu is available in the supplementary materials (S1).

### Low-GI Nutrigenetic diet

The low-GI nutrigenetic diet group had individualised dietary instructions based on their genetic results; examples of the advice given are found in Table 2. The gene variants were selected based on previous evidence of gene-diet interactions, in which a nutrition or exercise intervention was demonstrated to modify the effect of the variation, and which fulfilled the criteria described in [54]. The genetic results of each individual were then analysed for sensitivity to carbohydrates (utilising SNPs in *ACE, PPARG, TCF7L2, ADRB2* and *FABP2*) and saturated fats (*ADRB2, ADRB3, APOA2, FABP2, FTO, PPARG*). A score for both carbohydrate and saturated fat sensitivity was determined by utilising a point system, with the aggregate result being utilised. The base diet for each person was similar and was modified by refined carbohydrate content (maximum 10, 8%, or 6% total calories) and saturated fat content (maximum 10, 8%, or 6% total calories) according to the relevant sensitivity scores. Sample menus can be found in the supplementary files S2-S4. All subjects were advised to focus on whole-grain complex carbohydrates, as well as fruits and vegetables, as their primary sources of carbohydrates. Subject diets were also modified for some micronutrients and other macronutrients where the evidence is sufficient to deviate from the standard guidelines [54]. For example, individuals with a deletion allele for either *GSTM1* or *GSTT1* were recommended to increase their cruciferous vegetable intake [39], whilst those with a C allele for *CYP1A2* were recommended to limit their caffeine intake to < 200 mg/d [10].

**Table 2:** Examples of personalized recommendations given to the patients in Low-GI Nutrigenetic group in addition to base diet

**Personalized modifications to the standard guidelines based on DNA profile**	
**Variation in** ***ACE, PPARG, ADRB2*** **(Gln27Glu),** ***TCF7L2, FABP2***	Lower glycemic load (GL) diet, extra fiber, reduction of added sugars [3, 11, 25, 48]
**Variation in** ***LPL, FTO, APOA2, APOC3, ADRB2*** **(Arg16Gly),** ***ADRB3, PPARG, TCF7L2***	Restriction of saturated fats to no more than 16 g/day with concurrent increase in unsaturated fat consumption, such as olive oil [28]
**Variation in** ***GSTM1*** **and** ***GSTT1***	Ensure consumption of an adequate intake of cruciferous vegetables - 200 g five times per week [39]
**Variation in** ***GPX1***	Consume foods rich in selenium such as Brazil nuts, fresh fish, meat, wheat germs, brown rice, oats, and onion. In case of low plasma selenium, supplementation of 200 mcg/day was recommended [38]
**Variation in** ***TNF*** **and** ***IL6***	Increased consumption of omega-3 rich foods. Green tea, turmeric, ginger, rosemary, oregano were also recommended, along with supplementary omega 3 (1-2 g/day) [49]
**Variation in** ***MTHFR***	Increase consumption of folate-rich foods (dark leafy greens, asparagus, bean, peas, lentils, avocado, okra). Supplementation with 400mcg folate, 3 mg vitamin B6, 5 mg vitamin B12, 2.5 mg vitamin B2, 12 mg zinc, and 250 mg of TMG/betaine [9]
**Variation in** ***CYP1A2*** **and** ***EPHX1***	Increase consumption of antioxidants, such as grapes, blueberries, sweet potatoes and orange vegetables. Decrease in caffeine consumption. Decrease consumption of grilled meat and fish to 1–2 servings per week. [10, 35]
**Variation in** ***SOD2*** **and** ***CAT***	Increase antioxidant consumption through diet [12].
**Variation in** ***LCT***	Reduction of lactose, use lactose-free dairy. [44]
**Variation in** ***VDR***	Keep caffeine below 2 cups coffee/day. Increase dairy component of diet (yoghurt, cheese and low-fat milk). If required add supplement containing 800 IU vitamin D and 1300 mg Calcium. [51, 52]
**Variation in** ***HLA-DQ***	Check for symptoms of gluten intolerance – refer to medical doctor if necessary. [41]

### Exercise

Both groups were provided with general exercise advice and were asked to exercise for 30–45 min per day, 5 days per week. In both groups the exercise protocol for each person was carefully planned to avoid over-exertion in this overweight and generally sedentary cohort. The low-GI nutrigenetic group were given additional exercise advice, based on their results of six SNPs; *ACE, ADRB2, ADRB3, FTO, PPARG* and *TCF7L2*. As per the diet scoring, the results of each of these SNPs was combined, to give subjects guidance as to the volume of high, medium or low-intensity exercise recommended, total exercise duration was matched between the groups. Sample exercise plans are found in Supplementary Material file S5. Exercise adherence was based on questions at clinic visits.

### Dietary adherence

Patients visited the clinic every 2 weeks during the first 24 weeks for body measurements, and detailed dietary diaries were presented. Detailed diaries were maintained throughout the 24-week period, including the weighing of foods. All subjects received a menu plan with recipes. In addition, in the ketogenetic group, patients were taught how to test ketosis using Ketostix® (Ascensia Diabetes Care Holdings AG, Basel, Switzerland), measuring ketone bodies in the urine, which they did daily. Ketostix strips determine the presence of AA (acetoacetate). The end of the strip is passed through the urine stream and the colour then compared to the colour chart provided with the product. The scale is negative, trace, small, moderate, and large. Ketosis starts from “small”. In the 18-month follow-up period, patients presented at the clinic every 6 months for further body measurements and blood measurements, along with dietary diary assessment and exercise assessment (via diary and step counter). Dietary macronutrient composition was also tested.

### Subject testing

Cheek cell samples were taken in the clinic using two buccal swabs from the respective companies. The samples were sent by courier to the laboratory (Synlab Italia Srl, Monza, Italy). For DNA extraction swabs were added to 550 μl of sterile, nuclease-free H_2_O and DNA extracted with QIASYMPHONY DSP DNA Mini kit eluted in 100 μl liquid. The genotype analysis was done with MassARRAY system with iPLEX chemistry (ex-Sequenom now called Agena) on a 384 chip. Primers and PCR conditions were designed with Agena Assay Design Suite (ADS) software. Through this process, genetic information (Table 1) was determined.

Fasting venous blood samples were taken at baseline, 24-weeks and 104-weeks to determine total cholesterol (TC), high density lipoprotein (HDL) cholesterol, and fasting blood glucose (FBG). TC and HDL concentrations were measured using an enzymatic colorimetric method (CHOL-CHOD-PAP, HDL Homogenic Enzymatic reaction, Roche Diagnostic, Germany). FBG was determined using an enzymatic kit (Glucose GOD-PAP, Roche Diagnostic, Germany). Weight and height were also measured, and body mass index (BMI) was calculated by dividing each subject’s weight (kg) by the square of their height (m).

### Statistical analysis

All genotype distributions were tested for deviation from the Hardy-Weinberg equilibrium by a χ2 test with 1 df (*P* > 0.05). Means, standard deviations and 95% confidence intervals were calculated for test scores at baseline, 6 weeks, 12 weeks, 24 weeks and 2 years (104 weeks). Percentage weight and BMI change for each individual participant was calculated for all post-baseline time points. Percentage changes for each individual participant in TC, HDL and FBG were calculated between baseline and 2 years. Normality was determined utilising Shapiro-Wilks. Data were analysed using ANCOVA. To reduce the chances of a type-I error, significance was set at *p* < 0.001for secondary objectives [55]. Percentage change from baseline was calculated by subtracting baseline data from the measurement at a given time point, dividing by the baseline measurement, and multiplying by 100. Data were analysed using Microsoft Excel 15.29 (Microsoft Corporation, Redmond, WA, USA) and IBM SPSS Statistics 23 (IMB Corporation, Armonk, NY, USA). All data are reported as mean (95% CI) unless otherwise specified.

## Results

### Genotype frequencies

SNP minor allele and genotype frequencies were calculated from all subjects who completed the study and for which DNA data were available (*n* = 61). Genotype distributions did not deviate from Hardy-Weinberg expectations. Minor allele frequencies in our subjects were in close agreement with those listed for populations of European ancestry on dbSNP [56].

### Baseline phenotype

Table 3 illustrates the baseline characteristics of all subjects. There were no significant differences at baseline between the groups in terms of age, sex, body weight, or BMI. Except for one subject in the low-GI nutrigenetic group who was classified as overweight (BMI 25.1–30 kg/m^2^) all subjects were classified as obese (BMI > 30 kg/m^2^) according to BMI. There were no significant differences between the groups regarding mean total cholesterol or mean HDL at baseline. There were significant differences in terms of mean fasting blood glucose at baseline, with the low-GI nutrigenetic group having significantly lower (*p* < 0.0001) values.

**Table 3:** Baseline Characteristics of Subjects. All data are reported as mean (95% CI) unless otherwise specified

**Parameter**	**Ketogenic Diet**	**Low-GI Nutrigenetic Diet**	***p*-value**
Participants (n)	53	61	
Age (years) (±SD)	43.0 ± 7.2	42.0 ± 6.7	0.424
Female (%)	47.2%	55.7%	0.361
Baseline body weight (kg)	113.0 (109.4–116.6)	108.5 (104.4–112.6)	0.106
BMI (kg/m^2^)	37.2 (36.4–38.1)	37.0 (35.9–38.2)	0.789
Total Cholesterol (mg/dl)	245.6 (234.8–256.5)	242.0 (235.0–249.0)	0.56
HDL Cholesterol (mg/dl)	45.1 (43.4–46.8)	47.6 (46.4–48.8)	0.16
Fasting blood glucose (mg/dl)	120.5 (119.4–121.5)	105.7 (103.5–108.0)	**< 0.0001**

Data were analysed using ANCOVA, with significance set at *p* < 0.05 for primary observations and *p* < 0.001 for secondary observations

### Diet & nutrition adherence

All participants completed the 24-week study and 18-month follow up. In the first 4 weeks, all patients in the ketogenic group maintained ketosis. In the following weeks, 13 patients went out of a ketosis state; following dietary data analysis, it was determined that that 8 patients exceeded the amount of carbohydrates required to maintain ketosis, and 5 patients consumed higher protein than prescribed, which triggered gluconeogenesis. After re-adjusting the diet, these patients regained their ketosis state. Within the first year of follow up, 17 patients in the ketogenic group reported having deviated at least 3 times a month from the nutrition plan, consuming foods other than those prescribed. In the second year of follow-up, 24 patients were found to have diverged frequently from the nutrition plan due to special family events, social events, holidays, prolonged weekends and in some cases lack of motivation.

In the low-GI nutrigenetic group, during the 24-week diet phase, all patients successfully followed their individual diet plan. In follow up, 10 patients reported small deviations from the nutrigenetic nutrition plan, with these deviations relating to weddings, holidays, or anniversary events. The patients in the low-GI nutrigenetic group demonstrated greater adherence and consistency.

### Change in body mass and weight loss

**Fig. 1:**
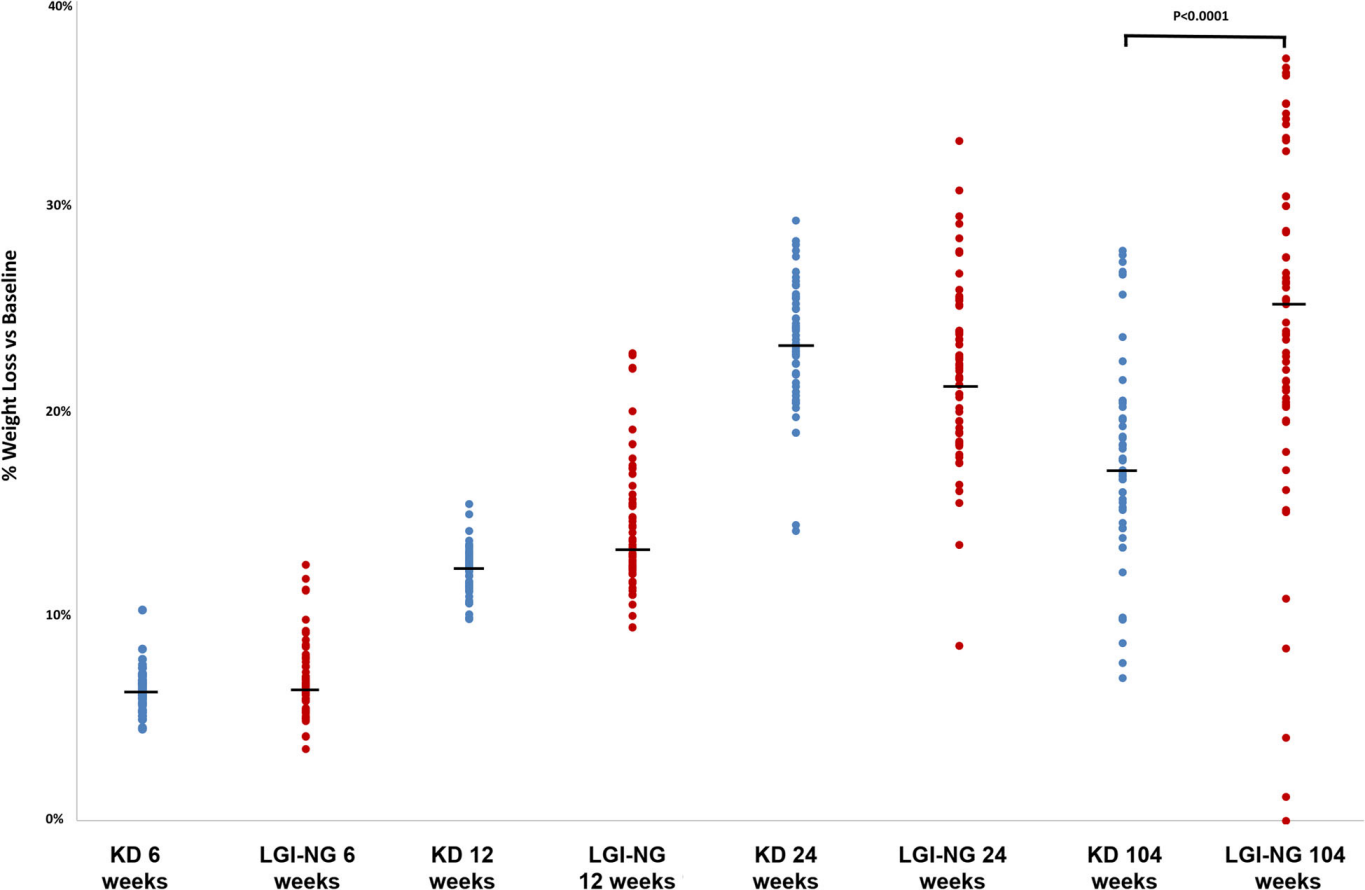
Percentage weight lost compared to baseline for each group (mean, 95% CI). At 104 weeks (2 years), the low-GI nutrigenetic group lost significantly more weight

Body mass changed in both diet groups, with similar weight loss at 6 and 12 weeks while at 24 weeks the ketogenic group had lost more weight compared to the low-GI nutrigenetic group. The ketogenic diet was associated with a 17.2% loss in body mass at 18-month follow up, which represented a significant (*p* < 0.0001) reduction. The low-GI nutrigenetic group was associated with a significant (*p* < 0.0001) reduction in body mass, with a mean reduction on 25.3%. When examining for differences between the groups, clear differences emerge at the two-year time point, with the low-GI nutrigenetic group continued to lose weight (p < 0.0001) while the ketogenic group had regained some weight compared to the 24-week time point (Table 4). Figure 1 details the percentage change in weight loss within each group.

**Table 4:** Body mass (kg) changes between diet groups

Time Point	Ketogenic group (*n* = 53)		Low-GI Nutrigenetic group (*n* = 61)		Significance
	Weight as % of baseline	Δ kg vs baseline (95% CI)	Weight as % of baseline	Δ kg vs baseline (95% CI)	
Baseline	100%		100%		
6 weeks	93.7	− 7.2 (− 7.5 to − 6.9)	93.3	−7.2 (−7.7 to −6.7)	1
12 weeks	87.9	−13.7 (−14.1 to − 13.3)	85.8	−15.5 (−16.5 to − 14.4)	0.0029
24 weeks	76.8	−26.2 (− 27.1 to −25.4)	78.4	−23.5 (− 25.1 to − 21.9)	0.0061
2 years	82.8	−19.4 (− 20.8 to − 18.0)	74.7	−27.5 (− 30.8 to − 24.3)	**< 0.0001**

Data were analysed using ANCOVA, with significance set at *p* < 0.05 for primary observations

### Health data

**Fig. 2:**
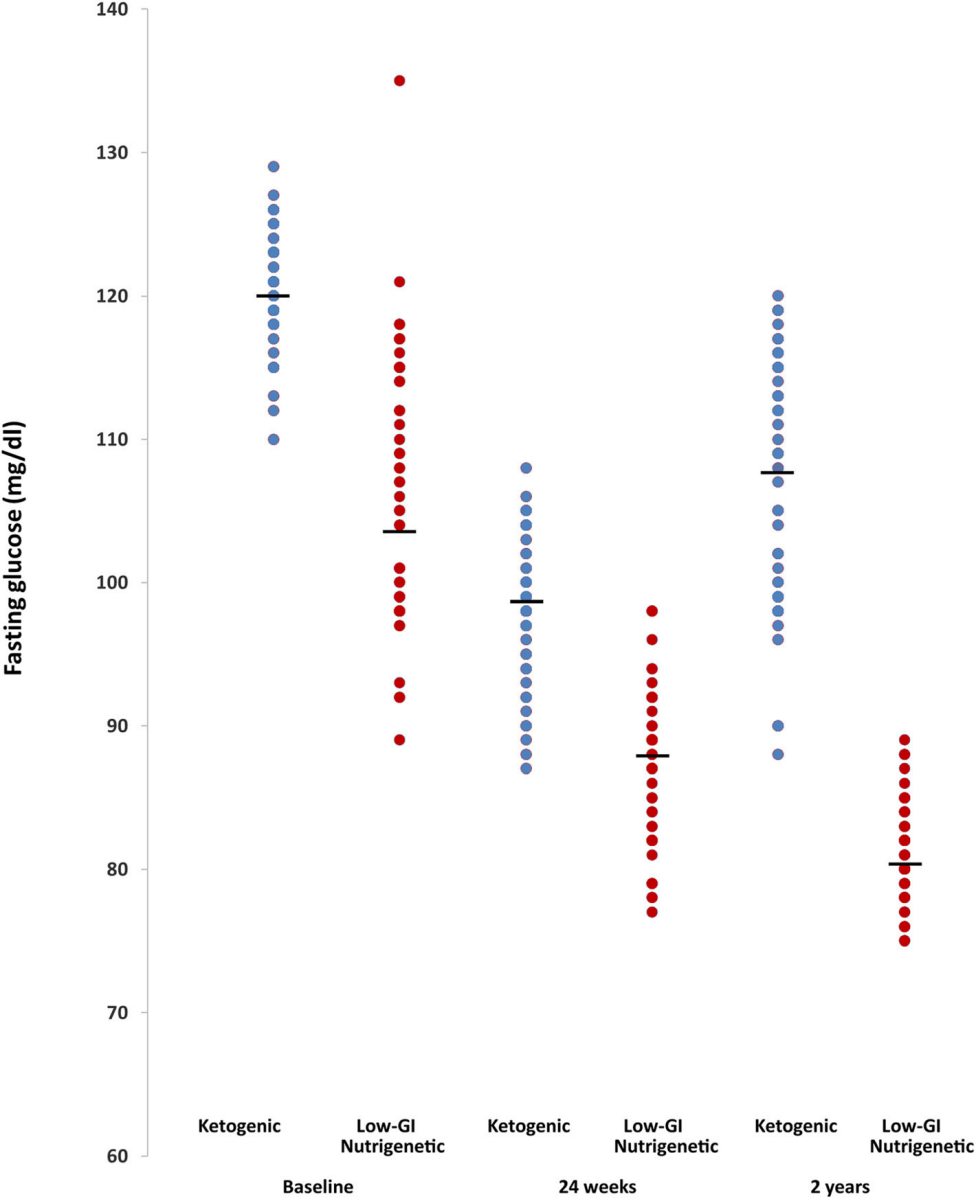
Individual fasting glucose (mg/dl) between diet groups

**Fig. 3:**
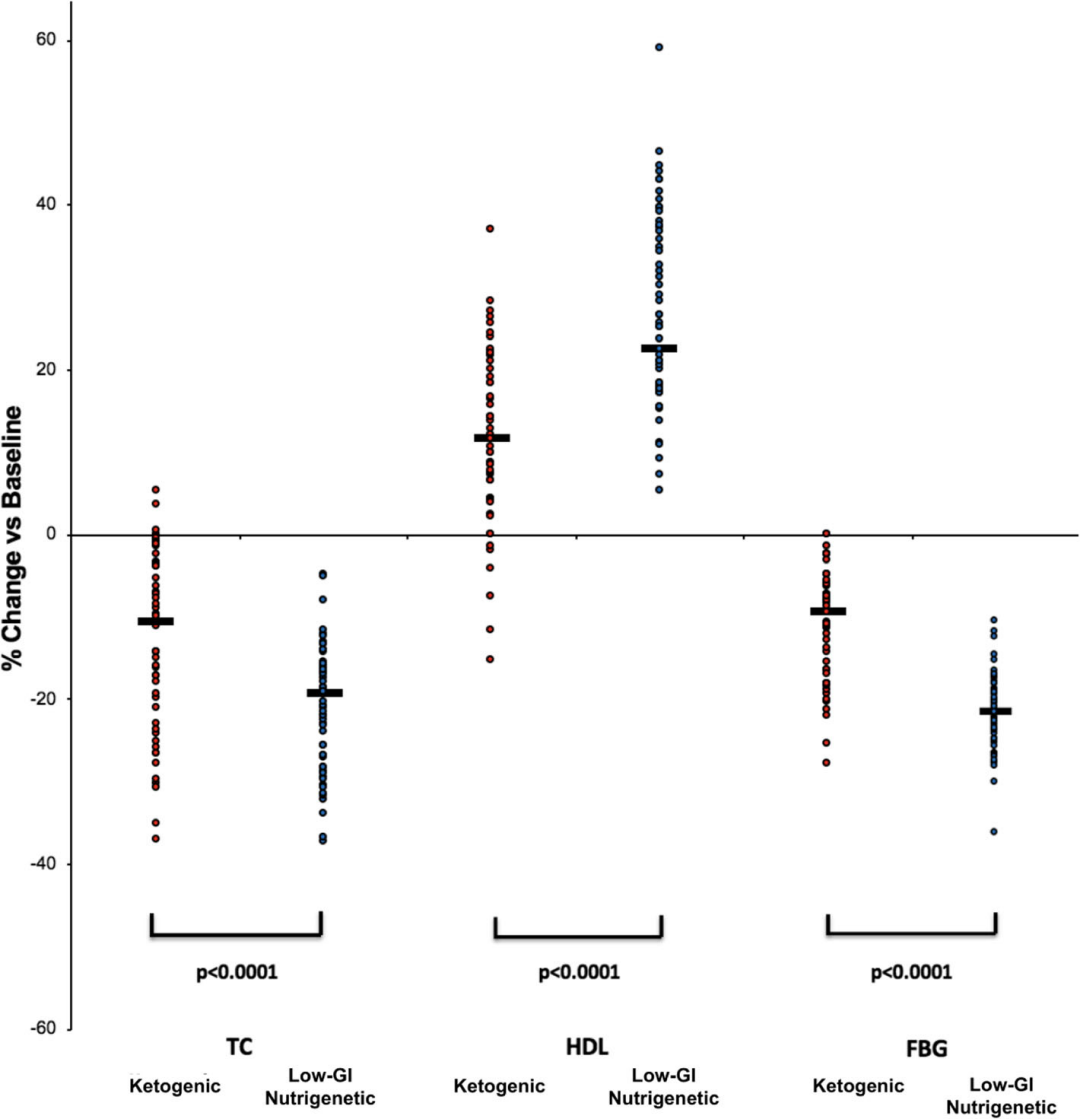
Percentage change from baseline for Total Cholesterol (TC), High Density Lipoprotein (HDL), and Fasting Blood Glucose (FBG) for both groups at two-year follow up

The results for metabolic health data are shown in Table 5 and Figs. 2 and 3. Here, we see that at 18-month follow up, the low-GI nutrigenetic group was associated with a significantly greater (*p* < 0.0001) decrease in both total cholesterol and fasting blood glucose when compared to the ketogenic diet group (Table 5). Similarly, the low-GI nutrigenetic group was associated with a positive HDL change (i.e. increase) to a significantly (p < 0.0001) greater extent than the ketogenic diet group (Table 5). There were significant differences in terms of mean fasting blood glucose at baseline, with the low-GI nutrigenetic group being associated with significantly lower values. However, there was considerable overlap at baseline between the two groups. This overlap became more marked at the 24-week point, with less difference in the mean values. The two groups diverged again at the two-year follow up point (Fig. 2). Figure 3 shows the individual participant metabolic health data. Finally, the low-GI nutrigenetic group was associated with a significantly (*p* < 0.0001) greater reduction in fasting glucose compared to the ketogenic diet group at the 18-month follow up point (Table 5).

**Table 5:** Differences in cardiometabolic parameters between both groups at different time points. All data are reported as mean (95% CI)

	**Total Cholesterol (mg/dl)**			**HDL Cholesterol (mg/dl)**			**Fasting blood glucose (mg/dl)**		
	Ketogenic	Low-GI Nutrigenetic	*P*	Ketogenic	Low-GI Nutrigenetic	*P*	Ketogenic	Low-GI Nutrigenetic	*P*
Baseline	245.6 (234.8–256.5)	242.0 (235.0–249.0)	0.56	45.1 (43.4–46.8)	47.6 (46.4–48.8)	0.16	120.5 (119.4–121.5)	105.7 (103.5–108.0)	**< 0.0001**
24 weeks	185.8 (181.4–190.2)	210.3 (205.5–215.0)	**< 0.0001**	54.1 (52.8–55.4)	55.2 (54.3–56.0)	0.182	98.2 (96.7–99.5)	87.0 (85.6–88.3)	**< 0.0001**
2 years	210.2 (204.7–215.7)	189.4 (187.7–191.1)	**< 0.0001**	49.8 (48.8–50.9)	59.5 (59.1–60.0)	**< 0.0001**	106.8 (104.4–109.1)	81.1 (80.2–81.8)	**< 0.0001**
Mean % Change at 2 years from baseline	− 13.0% (− 16.0 to − 10.0%)	−20.9% (− 22.8 to − 19.0%)	**< 0.0001**	11.6% (8.7–14.5%)	26.1% (23.3–29.1%)	**< 0.0001**	−11.3% (− 13.2 to − 9.4%)	−22.9% (− 24.3 to − 21.7%)	**< 0.0001**

Data were analysed using ANCOVA, with significance set at p < 0.05 for primary observations and *p* < 0.001 for secondary observations

## Discussion

The main findings of this study are (1) after 6 months diet the ketogenic group lost more weight compared to the LowGI/nutrigenetic group (26.2 kg vs 23.5 kg; *p* = 0.0061) (2). After 18-month follow-up the ketogenic group had regained some of the weight lost but the LowGI/nutrigenetics group continued to lose weight, at a slower rate (19.4 kg vs 27.5 kg; *p* < 0.0001). The LowGI/nutrigenetic group, after 18-month follow-up, also reported better results for total cholesterol, HDL and glucose. Also, though a few individuals of the LowGI/nutrigenetic group had regained some weight at 18-month follow-up (Fig. 1) all 61 were below the pre-diabetes blood glucose level of 100 mg/dl while only 16 of 53 of the keto group were below that level (Fig. 2).

These results mirror previous those of previous studies, such as those by Arkadianos et al. [5]. In the current study, these differences became more apparent over time; during the 18-month post-diet timeframe, particularly regarding changes in body weight. Indeed, up to the 24-week time point, there were significant differences between the diet groups in terms of body weight change and biomarker improvement in favour of the ketogenic group.

Long term maintenance of weight loss requires permanent lifestyle changes in exercise and eating habits. These changes need to be significant but not necessarily radical or unachievable if planned over several years of gradual but sustainable weight loss. Nutrigenetic modifications, as part of personalized nutrition (PN) add a small part to the overall diet, with an obvious question being whether it improves adherence to healthy nutrition. Nutrigenetics includes advice dependent on genetic results and following the straight-forward gene-diet advice the aim is that the overall eating pattern will improve. Nutrigenetic PN does not create complex personalized diets, merely slight differences between diet types.

One possibility for this enhanced long-term weight loss, combined with improvements in markers of metabolic health, is that healthy eating compliance is greater when nutrigenetics is used to modify the standard nutrition guidelines at the end of the dieting phase. The dietary adherence data in this study agrees with this possibility, as does previous research in personalized nutrition. Nielsen and El-Sohemy [57] reported that participants tend to find genetically tailored nutritional advice useful. A subsequent study by the same authors [8] reported that personalized dietary advice based on a person’s genetic makeup improves eating habits compared to current ‘one-size-fits-all’ dietary recommendations. The authors reported that subjects who received DNA-based dietary advice started to show improvements to their diets after 3 months, with the changes becoming more apparent after 12 months. A recent randomised controlled trial of nutrigenomics-guided lifestyle intervention reported “Weight management interventions guided by nutrigenomics can motivate long-term improvements in dietary fat intake above and beyond gold-standard population-based interventions.” [6].

Some studies exploring the genetic risk of common diseases have found varying results, with some reporting that knowledge of genetic risk score has little impact on behaviour [58]. Key differences with personalized nutrigenetics studies, compared to genetic risk studies, include that (a) genetic information is linked only to nutrient/lifestyles requirements and is not explicitly linked to disease risk and (b) the information given to individuals includes precise advice on increasing or decreasing specific food groups. Thus, adherence to a dietary/lifestyle intervention appears to be more successful in nutrigenetics compared to a genetic disease risk score, which often do not come with personalised nutrition guidelines to mitigate any increased risk. As a result, there is increasing evidence that one of the main benefits with nutrition and genetics is that of behavioural change [59].

It is important to note some potential limitations to this current study. The mechanisms underpinning the enhanced weight loss and health improvements in the low-GI nutrigenetic group are unclear; it may be potentially due to dietary adherence, or specific biological mechanisms, which were not tested. It is also possible that the effects were placebo or expectancy mediated in nature. Additionally, the lead study author was not blinded to the results of each individual’s genotype results, which may have affected the study outcome.

Another limitation is that in this study the associations observed with the low GI/nutrigenetic diet were only in comparison to a ketogenic diet, it would have been ideal to have other groups including lowGI only, without nutrigenetics – but that wasn’t feasible with the resources available and we had showed previously that a lowGI/nutrigenetics diet gave better long-term results to lowGI only [5].

The choice of a ketogenic diet as the control diet has the potential to alter the results; a ketogenic diet can be difficult to adhere to for certain individuals [60]. A ketogenic diet may also disturb basal metabolic processes leading to adverse reactions when returning to standard nutrition. Additionally, fasting blood glucose was significantly lower at baseline in the low-GI nutrigenetic group when compared to the ketogenic diet. The low-GI nutrigenetic group lowered their fasting blood glucose to a greater extent in this study, and the baseline differences may have contributed to this variation between groups. It is not clear why these baseline differences were present, but they overlapped.

An additional limitation is that the population used in this study were almost exclusively obese; it is not clear whether such a lifestyle modification would be effective in non-obese, but overweight, individuals – although other studies demonstrated an improved healthy-eating index in such people [61]. However, despite these issues, the study addressed an important unmet need to generate real world data; a common issue with clinical trials is that participant behaviour may be altered simply by being part of a study [62]. It has been increasingly recognized that such data in real world settings is needed to improve health outcomes [63]. Thus, we believe that the present study does accurately represent the real-world, in which high-risk individuals were given a dietary intervention in order to improve health. Study subjects were European Caucasians; ethnicity is a known potential modifier of gene-diet interactions, so it’s not clear whether the findings of this study would hold true for other populations, although the majority of the variants utilised here are functional—i.e. they directly affect the protein—and so their effect should be the similar regardless of ethnicity. Further research in this area should examine the use of a low-GI nutrigenetic personalized nutrition to reduce the risk of developing obesity or metabolic syndromes in healthy, non-obese subjects, as well as replicating the results of this study in populations of non-European ethnicity. Finally, this study utilised a low-GI, nutrigenetic diet, and compared the outcomes of this diet to a ketogenic diet. As the ketogenic diet group did not undergo genetic testing, and have their nutritional intervention tailored to their genetic results, a next study should look at the addition of nutrigenetic advice after the 24-week ketogenic diet, to see if the benefits of the ketogenic diet are maintained in the long term, especially as after the 24-week diet and benefits were mostly greater in the ketogenic group.

## Conclusions

The results of this study suggest that a 24-week, ketogenic diet was superior to a low-GI nutrigenetic diet at improving weight loss and health markers compared to baseline upon completion of the dietary intervention, but at 18-month follow up the low-GI nutrigenetic group fared better. These findings suggest that despite the better results in the ketogenic group following a 24-week dietary intervention, over longer periods the low-GI nutrigenetic diet may be useful in the treatment of both obesity and altered blood markers of metabolic health, and that these benefits appear to be maintained following the completion of a dietary intervention, an effective aid in long term lifestyle changes leading to sustained weight loss and health improvements.

## Supplementary information

**Additional file 1.** S1. Sample Ketogenic Diet meal plan. S2. Sample Nutrigenetic (Low Carbohydrate) meal plan. S3. Sample Nutrigenetic (Mixed Diet) meal plan. S4. Sample Nutrigenetic (Low Fat) meal plan. S5. Sample exercise plans.

## Data Availability

The datasets used and/or analysed during the current study are available from the corresponding author on reasonable request.

## Abbreviations

BMI: Body mass index
EU: European Union
FBG: Fasting blood glucose
GL: Glycaemic load
HDL: high-density lipoprotein cholesterol
Low-GI: Low-glycaemic index
LowGI/NG: Low-Glycaemic Index nutrigenetic diet
PN: Personalized nutrition
RDA: Recommended daily allowance
SNP: SINGLE Nucleotide Polymorphism
TC: Total cholesterol
