# Supplementary material for: A comparison of a ketogenic diet with a LowGI/nutrigenetic diet over 6 months for weight loss and 18-month follow-up
Source: BMC Nutr. 2020 Sep 24;6:53. doi: 10.1186/s40795-020-00370-7 (PMC7513277; doi:10.1186/s40795-020-00370-7)
Supplement: Supplementary file 1 — Additional file 1. S1. Sample Ketogenic Diet meal plan. S2. Sample Nutrigenetic (Low Carbohydrate) meal plan. S3. Sample Nutrigenetic (Mixed Diet) meal plan. S4. Sample Nutrigenetic (Low Fat) meal plan. S5. Sample exercise plans. [file 40795_2020_370_MOESM1_ESM.docx]

**S1 – Sample Meal Plan for Ketogenic Diet**

Meal 1

**Keto muffin**

Serving size 2 portions

- 4 cherry tomatoes
- ¼ cup red onion, chopped
- 1 cup mixed greens (Spinach is great too!)
- 8 egg yolks
- ⅓ cup bacon, crumbled
- 1 ⅕ cup cheddar cheese, shredded
- 3 tbsp. unsweetened almond milk (optional)
- ½ tsp garlic salt

Meal 2

**Keto salad**

**Ingredients for 1 portion**

- 2 cherry tomatoes
- ½ avocado
- 1 hardboiled egg
- 2 cups mixed green salad
- 2 oz. chicken breast, shredded
- 1 oz. feta cheese, crumbled
- ¼ cup cooked bacon, crumbled

Meal 3

Pizza with mozzarella and pepperoni

Ingredients for 1 portion

- Slices (34 g), sandwich sliced pepperoni
- 2 tbsp. pizza sauce
- 3 oz. fresh mozzarella
- 1 tbsp. fresh oregano

Meal 4

Chocolate keto desert serving size 2 balls

Ingredients for 6 balls

- ¼ cup unsweetened cocoa powder (or cacao powder)
- 5 tbsp. natural chunky peanut butter
- 6 tbsp. shelled hemp seeds
- ½ cup, coconut oil
- 2 tbsp. 10% heavy cream
- 1 tsp. vanilla extract
- 2 tbsp, Stevia or xylitol
- 4 tbsp. unsweetened coconut flakes

**S2 – Sample Meal Plan for Low Carbohydrate**

**Breakfast (350 kcal)**

Boiled egg and toast with fruit

1 hard-boiled egg with 2 slices whole bread

2 slice wholewheat bread

1 fruit- medium orange

**Lunch (550 kcal)**

Chicken, bean and arugula salad

2 slices whole wholewheat bread

1 medium apple

**Dinner (450 kcal)**

Three bean salad

Chop 3 spring onions, 5 cherry tomatoes and 1 green pepper.

Mix with 3tbsp each of red kidney beans, chick peas and cannelini beans and 2tbsp olive oil

1 wholewheat bread roll

**Snack (250 kcal)**

Chop 1 kiwi fruit or 1 small banana and 20 almonds and 100g greek yogurt 2% fats

**Total kcal 1600**

**Glycemic load value per day 65**

**S3 – Sample Meal Plan for Mixed Diet**

**Breakfast**

Partially skimmed milk 250ml + 40g of breakfast cereals (oatmeal) or simple biscuits

dried + honey or jam 25g

Note: if lactose intolerant, lactose free milk recommended

**Lunch**

Wholegrain Pasta (80g) with tuna (50g) and tomatoes sauce

green vegetables on desire

+ 150g of fresh seasonal fruit

**Dinner**

Baked turkey 150g

Wholegrain rice boiled 100g

Vegetables on desire

150g fresh seasonal fruit

**Snacks**: A portion of fresh seasonal fruit (150g) or a low-fat fruit yoghurt

Seasoning throughout the day:

40g of extra virgin olive oil (8 teaspoons)

**S4 – Sample meal plan for Low Fat Diet Group**

**Breakfast**

• Oatmeal (about ¾ cup)
• small Banana sliced
• Orange Juice 100 ml
• unsweeted Coffee or tea optional

## **Lunch**

 • 1/2 tuna salad sandwich (with lettuce, tomato, and 2 tbs olive oil )
 • Cup of minestrone-vegetables soup
 • 1 Apple

**Dinner**

 • Salmon with pineapple salsa
 • 6 boiled asparagus
 • Wholegrain rice (about 1/2 cup) 100g boiled
 • dessert homemade peach ice cream prepared with fat free milk (0.1g of fats per serving and 58 calories)

**Snacks 1**

Nuts 'n’ Berries

2/3 cup blueberries sprinkled with 1 tablespoon slivered almonds

**Snacks 2**

Baked Apple

1 small apple, cored, filled with 1 teaspoon brown sugar and 1 sprinkle cinnamon, baked until tender

Total calories 1600 (Less than 16g of saturated fat per day)

**S5 – Physical activity tables**

Both groups were given general exercise advice, and asked to exercise for 30-45 minutes, 5 times per week. Additionally, the nutrigenetic group were given genetically matched exercise guidelines:

**Table S5.1 – A sample exercise plan for low intensity, long duration exercise.**

| Low intensity, long duration   - 40-60% of your maximum heart rate - There is no noticeable change in breathing patterns - Does not induce sweating unless it's a hot, humid day. - You can easily have a sustained conversation and can even sing | | | |
| --- | --- | --- | --- |
| Gym | **METs** | **General** | **METs** |
| Cyclette 50 watt | 3 | Walking slowly (< 3 kmh) | 2 |
| Running machine - slow | 3 | Stretching, Hatha yoga | 2,5 |
| Rowing machine, 50 watt | 3,5 | Playing guitar / piano | 2,5 |
| Ellipse - light | 3,5 | Walking downhill (4 kmh) | 3 |
| Acquafitness | 4 | Slow dance (waltz, mambo, tango) | 3 |
| Tai Chi, yoga etc | 4 | Medium bicycle (< 16 kmh) | 4 |
| Step aerobics – slow | 4 | Gardening | 4 |
| Stepper – slow | 4 | Playing the drums | 4 |
| Ellipse Cross - slow | 4 | Tai Chi | 4 |
|  |  | Fast dancing (folk, country, polka) | 4,5 |
|  |  | Golf | 4,5 |

**Table S5.2 - A sample exercise plan for medium intensity, medium duration exercise**

| Medium intensity & duration   - 70% of your maximum heart rate - Breathing becomes deeper and more frequent. - Will break a sweat after performing the activity for about 10 minutes - You can carry on a conversation but not sing | | | |
| --- | --- | --- | --- |
| Gym | **METs** | **General** | **METs** |
| Cyclette, 100 watt | 5,5 | Hiking | 6 |
| Weight lifting vigorous effort | 6 | Walking uphill (6 kmh) | 6 |
| Cyclette, 150 watt | 7 | Wood & grass cutting | 6 |
| Rowing machine, 100 watt | 7 | Bicycling 10-11.9 mph, slow, light effort | 6 |
| Aerobics | 7 | Jogging | 7 |
| Running machine- jogging | 7 | Tennis | 7 |
| Ellipse - medium | 7 | Ski | 7 |
| Step aerobics – medium | 7,5 | swimming laps, freestyle, slow, light effort | 7 |
| Stepper – medium | 7,5 | Running (8 kmh) | 8 |
| Ellittica Cross - medium | 7,5 | Rock climbing | 8 |
| Spinning – medium | 8 | Fast walking (8 kmh) | 8 |
|  |  | Basketball or volleyball matches | 8 |
|  |  | bicycling, 12-13.9 mph, moderate effort | 8 |
|  |  | Mountain bike | 8,5 |

**Table S5.3 - A sample exercise plan for high intensity, short duration exercise**

| High intensity, short duration   - 80-85%% of your maximum heart rate - Breathing is deep and rapid - Will break a sweat after 3-5 minutes. - You can only talk in short phrases. | | | |
| --- | --- | --- | --- |
| Gym | **METs** | **General** | **METs** |
| Cyclette, 200 watt | 9 | Orienteering | 9 |
| Weight lifting vigorous effort | 9 | Running (10 kmh) | 10 |
| Cyclette, 250 watt | 11 | Kickboxing, judo, karate | 10 |
| Aerobica - rapid | 12 | Football or Rugby match | 10 |
| Rowing, 200 watt | 12 | Skipping | 10 |
| Running machine - running | 13 | swimming, crawl, fast (75 yards/minute) | 10 |
| Ellipse - rapid | 13 | bicycling, 14-15.9 mph, fast, vigorous effort | 10 |
| Step aerobics – fast | 13,5 | swimming, butterfly, general | 11 |
| Stepper – strong | 14 | In line skating | 12 |
| Ellipse Cross - strong | 14 | bicycling, 16-19 mph, racing/not drafting or >19 mph drafting, very fast, racing general | 12 |
| Spinning – strong | 16 | Running (13 kmh) | 13,5 |
|  |  | bicycling, >20 mph, racing, not drafting | 16 |

**Table S5.4 - Supporting References for exercise advice:**

| ACE | Dengel DR, Brown MD, Ferrell RE et al. Exercise-induced changes in insulin action are associated with ACE gene polymorphisms in older adults. *Physiol Genomics*. 2002. 11(2):73-80. |
| --- | --- |
| LPL | Garenc C, Perusse L, Bergeron J et al. Evidence of LPL gene-exercise interaction for body fat and LPL activity: the HERITAGE Family Study. J Appl Physiol. 2001. 91(3):1334-40. |
| VDR | Grundberg E et al (2004). Genetic variation in the human vitamin D receptor is associated with muscle strength, fat mass and body weight in Swedish women. Eur J Endocrinol. 150(3):323-328. |
| ADRB2 | Macho-Azcarate T et al (2003). Basal fat oxidation and after a peak oxygen consumption test in obese women with a beta2 adrenoceptor gene polymorphism. J Nutr Biochem. 14(5):275-279. |
| ADRB2 | Ruiz JR et al (2011). Role of β₂-adrenergic receptor polymorphisms on body weight and body composition response to energy restriction in obese women: preliminary results. Obesity (Silver Spring). 19(1):212-215. |
| ADRB3 | Ann Nutr Metab. 2009;54(2):104-10. doi: 10.1159/000209268. Epub 2009 Mar 19. Influence of Trp64Arg polymorphism of beta 3-adrenoreceptor gene on insulin resistance, adipocytokines and weight loss secondary to two hypocaloric diets. de Luis DA1, González Sagrado M, Aller R, Izaola O, Conde R. |
| ADRB3 | J Physiol Anthropol. 2010;29(4):133-9. The Trp64Arg polymorphism of the beta3-adrenergic receptor gene is associated with weight changes in obese Japanese men: a 4-year follow-up study. Yamakita M1, Ando D, Tang S, Yamagata Z. |
| ADRB2 | Obesity (Silver Spring). 2011 Mar;19(3):595-603. doi: 10.1038/oby.2010.224. Epub 2010 Oct 7. Lifestyle and socioeconomic-status modify the effects of ADRB2 and NOS3 on adiposity in European-American and African-American adolescents. Lagou V1, Liu G, Zhu H, Stallmann-Jorgensen IS, Gutin B, Dong Y, Snieder H. |
| ADRB2 | J Nutr. 2003 Aug;133(8):2549-54. Obesity risk is associated with carbohydrate intake in women carrying the Gln27Glu beta2-adrenoceptor polymorphism. Martínez JA, Corbalán MS, Sánchez-Villegas A, Forga L, Marti A, Martínez-González MA. |
| PPARG | Nelson TL, Fingerlin TE, Moss LK, Barmada MM, Ferrell RE, Norris JM. (2007). Association of the peroxisome proliferator-activated receptor gamma gene with type 2 diabetes mellitus varies by physical activity among non-Hispanic whites from Colorado. Metabolism. 2007 Mar;56(3):388-93 |
| PPARG | 52. Ruchat SM, Rankinen T, Weisnagel SJ, Rice T, Rao DC, Bergman RN, et al. Improvements in glucose homeostasis in response to regular exercise are influenced by the PPARG Pro12Ala variant: results from the HERITAGE Family Study. Diabetologia. 2010;53(4);679-89. |
| pparg | Lindi VI, Uusitupa MIJ, Lindstrom J, et al. Association of the Pro12Ala polymorphism in the PPAR-{gamma}2 gene with 3-year incidence of type 2 diabetes and body weight change in the Finnish diabetes prevention study. Diabetes 2002; 51:2581–6 |
| pparg | Ostergard T, Ek J, Hamid Y, et al. Influence of the PPAR-gamma2 Pro12Ala and ACE I/D polymorphisms on insulin sensitivity and training effects in healthy offspring of type 2 diabetic subjects. Horm Metab Res 2005; 37:99–105. |
| TCF7L2 | Diabetes. 2010 Mar;59(3):747-50. doi: 10.2337/db09-1050. Epub 2009 Dec 22. Gene variants of TCF7L2 influence weight loss and body composition during lifestyle intervention in a population at risk for type 2 diabetes. Haupt A1, Thamer C, Heni M, Ketterer C, Machann J, Schick F, Machicao F, Stefan N, Claussen CD, Häring HU, Fritsche A, Staiger H. |
| TCF7L2 | Am J Clin Nutr. 2009 Dec;90(6):1502-8. doi: 10.3945/ajcn.2009.28379. Epub 2009 Oct 28. Effects of TCF7L2 polymorphisms on glucose values after a lifestyle intervention. Bo S1, Gambino R, Ciccone G, Rosato R, Milanesio N, Villois P, Pagano G, Cassader M, Gentile L, Durazzo M, Cavallo-Perin P. |
